# Transient cortical blindness following coronary angiography: a case report

**DOI:** 10.3389/fcvm.2026.1774967

**Published:** 2026-02-10

**Authors:** Ying Cai, Shengyu Huang, Yuling Jing, Ruijie Ren, Zaiyong Zheng

**Affiliations:** 1 Cardiovascular Center, Chongqing Hospital of Jiangsu Province Hospital, Chongqing, China; 2 Department of Cardiology, The Affiliated Hospital, Southwest Medical University, Luzhou, China; 3 Department of Ultrasound, Chongqing Hospital of Jiangsu Province Hospital, Chongqing, China; 4 Department of Cardiology, Panzhihua Central Hospital, Panzhihua, China

**Keywords:** case report, contrast medium, coronary angiography, cortical blindness, occipital lobe

## Abstract

Transient cortical blindness is an extremely rare and reversible neurological complication following angiographic procedures and is thought to be related to transient dysfunction of the occipital cortex. We report a 62-year-old woman with hypertension who underwent coronary angiography using 40 mL of the iso-osmolar contrast medium iodixanol. No significant coronary stenosis was identified. Approximately 10 min after contrast medium injection, she developed rapidly progressive bilateral visual loss, which progressed to complete blindness, accompanied by dizziness but without other neurological deficits. She remained alert, with preserved pupillary light reflexes and a normal ophthalmologic examination. Emergent cranial CT and MRI, performed approximately 25 min after contrast injection, showed no evidence of acute infarction or hemorrhage. Based on the close temporal association with contrast exposure and the exclusion of other causes, contrast-induced transient cortical blindness was considered. With supportive treatment, her vision gradually recovered and returned nearly to normal within one week. No abnormalities were observed at the 3-month follow-up.

**Conclusion:**

Even iso-osmolar contrast agents such as iodixanol may induce transient cortical blindness. Further studies are needed to clarify the underlying mechanisms.

## Introduction

Coronary angiography is widely used for the diagnosis of coronary artery disease and is generally considered a safe procedure. Neurological adverse reactions related to contrast media have occasionally been reported. Among them, transient cortical blindness is extremely rare, with an incidence of less than 0.1% following coronary angiography (1). Previous studies suggest that high-osmolar contrast agents are more frequently associated with this complication. Here, we report a case of transient cortical blindness occurring after coronary angiography using the iso-osmolar contrast medium iodixanol.

## Case presentation

A 62-year-old woman was admitted with a 2-year history of recurrent chest pain. She had a 3-year history of hypertension treated with amlodipine, with blood pressure well controlled at approximately 110–120/70–80 mmHg. Laboratory testing showed mildly abnormal lipid parameters, with an LDL-C level of 1.72 mmol/L, a triglyceride level of 2.01 mmol/L, and an estimated glomerular filtration rate of 88.0 ml/min/1.73 m^2^. Electrocardiography demonstrated sinus rhythm with flattened T waves. Transthoracic echocardiography revealed mild tricuspid and pulmonary regurgitation, impaired left ventricular diastolic function, and preserved systolic function. Carotid ultrasonography showed carotid atherosclerotic plaques. Coronary angiography was planned because of suspected coronary artery disease.

## Angiographic procedure

Coronary angiography was performed via the radial artery using 40 ml of the iso-osmolar contrast agent iodixanol. The procedure was uneventful, and no significant stenosis was observed in either coronary artery. During the procedure, blood pressure transiently increased to 145/78 mmHg, with a heart rate of 62 beats per minute.

**Figure 1:**
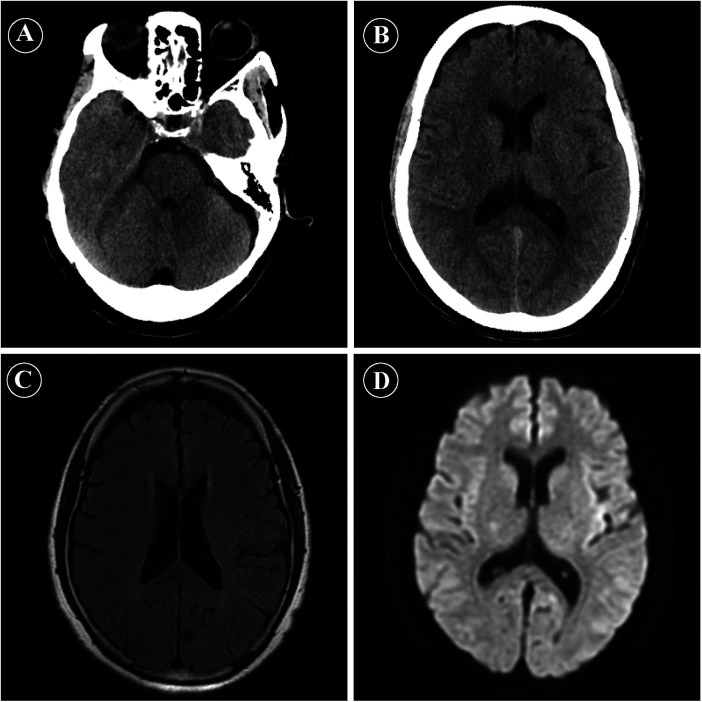
Emergency neuroimaging findings after coronary angiography. **(A,B)** Non-contrast cranial computed tomography (CT) images obtained shortly after symptom onset, showing no evidence of intracranial hemorrhage or acute ischemic lesions. **(C)** Axial fluid-attenuated inversion recovery (FLAIR) magnetic resonance imaging (MRI) showing no abnormal signal intensity in the bilateral occipital lobes or other brain regions. **(D)** Diffusion-weighted imaging (DWI) revealing no diffusion restriction, effectively excluding acute cerebral infarction.

Approximately 10 min after completion of the procedure, the patient developed rapidly progressive bilateral visual loss, which progressed to complete blindness within several minutes. She also reported dizziness but denied limb weakness, speech difficulties, or seizures. On examination, she was alert and oriented, with intact pupillary light reflexes and no focal neurological deficits. Funduscopic examination was normal. Cranial CT and MRI (Figure 1) were performed approximately 8 min after symptom onset and demonstrated no evidence of intracranial hemorrhage, acute ischemic lesions, or abnormal signal changes in the occipital lobes. Given the close temporal relationship with contrast administration and the exclusion of other causes, a diagnosis of contrast-induced transient cortical blindness was considered.

## Treatment and follow-up

The patient received supportive treatment, including hydration and symptomatic therapy. Glucocorticoids, nimodipine, betahistine, and mannitol were administered according to local clinical practice. Her visual function gradually improved and returned to near normal within one week. No recurrence of visual disturbances was observed during the 3-month follow-up.

## Discussion

Transient cortical blindness is a reversible visual disturbance considered to represent a specific manifestation of contrast-induced encephalopathy, associated with transient dysfunction of the occipital cortex. In the present case, the patient developed acute bilateral visual loss shortly after coronary angiography while remaining alert, with preserved pupillary reflexes and normal neuroimaging findings. Her symptoms resolved completely with supportive treatment. Similar cases have been reported not only after coronary angiography but also following other endovascular procedures (2). Cortical blindness is a diagnosis of exclusion. Typical features include: (1) transient visual impairment with preserved pupillary reflexes, absence of ocular pathology; (2) neuroimaging findings that exclude acute stroke. In some patients, transient occipital abnormalities may be detected on imaging. In our patient, advanced cerebral angiography such as CTA, MRA or DSA were not performed. Nevertheless, based on close temporal relationship with contrast exposure, clinical feature and the absence of acute abnormalities on non-angiographic cranial imaging, contrast agent-related transient cortical blindness was considered the most likely diagnosis. In the present case, cranial CT and brain MRI including DWI were obtained approximately 25 min after contrast injection. Although no diffusion restriction was observed, it should be noted that diffusion-weighted imaging may be falsely negative in the hyperacute phase, particularly in posterior circulation ischemia, which represents a potential limitation of early neuroimaging in this setting (3).

Contrast-induced encephalopathy is a rare and usually reversible complication, although permanent neurological sequelae have been reported in some cases (4). Its clinical manifestations are variable and may include headache, confusion, memory impairment, visual and speech disturbances, seizures, hemiparesis, and even coma (4). The underlying mechanism is not fully understood but is thought to involve neurotoxic effects of contrast media. Factors such as high contrast concentrations, blood–brain barrier (BBB) dysfunction, and impaired renal clearance of contrast agents may contribute to its development. Transient cortical blindness is regarded as a distinct manifestation within the spectrum of contrast-induced encephalopathy, characterized predominantly by transient visual impairment.

As the primary visual cortex is located in the occipital lobe, most reported cases of cortical blindness involve conditions that affect occipital lobe function (5). For example, contrast retention in the occipital cortex has been described in some patients. Cortical blindness has also been reported in children with occipital bone fractures, suggesting that different forms of occipital lobe injury or dysfunction may lead to this condition (6). In the present case, however, the close temporal association with contrast administration strongly supports contrast-induced cortical blindness as the most plausible explanation.

The occipital lobe is supplied by the vertebrobasilar–posterior cerebral artery system, which may be particularly sensitive to abrupt changes in cerebral perfusion or vascular regulation. Accordingly, cortical blindness has been reported following cervical spine injuries affecting vertebral artery flow, accidental intra-arterial infusion of saline via the brachial artery, or inadvertent injection of contrast near the vertebral artery through the internal mammary artery (7–9). Other reported associations include hepatic encephalopathy after Transhepatic Intrajugular Portosystemic Shunt (TIPS) (10), microemboli in pulmonary embolism (11), and ozone disc therapy in patients with patent foramen ovale (12), major cardiac surgery (13), postpartum hemorrhage (14), and respiratory failure (15). A previous report also described cortical blindness after resection of a pineal region meningioma, in which venous congestion of the occipital cortex was considered a contributing factor (16).

High-osmolar contrast agents have traditionally been associated with a higher risk, however, increasing evidence indicates that iso-osmolar and low-osmolar agents may also induce this condition, suggesting a role for intrinsic neurotoxicity (4). Iodinated contrast media used in coronary angiography and contrast CT are the most commonly implicated. In contrast, cortical blindness following gadolinium-based MRI contrast agents is rarely reported, possibly reflecting differences in acute neurotoxic potential. Although contrast agents typically do not cross an intact blood–brain barrier (BBB), age-related BBB dysfunction increase susceptibility. Other neurotoxic substances, such as vincristine, and metabolic disturbances such as hyperammonemia, have also been associated with transient cortical blindness (17).

Contrast-induced cortical blindness represents one subtype of cortical blindness. Given its distinctive mechanism and the widespread use of contrast media, clearer documentation of contrast-related cases and further investigation into potential risk factors are warranted. In patients presenting with acute visual loss suggestive of cortical blindness, prompt neuroimaging remains essential to exclude acute cerebrovascular events and to guide appropriate management.

## Conclusion

Contrast-induced transient cortical blindness is a rare but reversible complication following coronary angiography. Its occurrence may be related to transient occipital cortical dysfunction involving occipital visual cortex. Greater awareness of this condition may help clinicians avoid misdiagnosis and unnecessary interventions.

## Data Availability

The original contributions presented in the study are included in the article/Supplementary Material, further inquiries can be directed to the corresponding author.

## References

[1] Damavandi PT Cali D Negro G Girombelli A Lattanzi S. Contrast medium-induced transient cortical blindness: a systematic review of the literature. J Vasc Interv Radiol. (2024) 35(10):1439–46.e16. 10.1016/j.jvir.2024.06.007 38906244

[2] Liang KW Lin HY Cheng KL Wang B Huang HH. Transient cortical blindness following transarterial embolization for shoulder adhesive capsulitis. J Vasc Interv Radiol. (2024) 35(10):1565–7. 10.1016/j.jvir.2024.04.029 38901490

[3] Edlow BL Hurwitz S Edlow JA. Diagnosis of DWI-negative acute ischemic stroke: a meta-analysis. Neurology. (2017) 89(3):256–62. 10.1212/WNL.0000000000004120 28615423 PMC5513816

[4] Zhang Y Zhang J Yuan S Shu H. Contrast-Induced encephalopathy and permanent neurological deficit following cerebral angiography: a case report and review of the literature. Front Cell Neurosci. (2023) 16:1070357. 10.3389/fncel.2022.1070357 36687520 PMC9847581

[5] Raval M Trivedi J Curry I Sanghvi L Patel S Alexandrov P Transient cortical blindness after cardiac catheterization: a case report and review of possible neurological etiologies. J Community Hosp Intern Med Perspect. (2023) 13(6):24–28. 10.55729/2000-9666.1249 38596558 PMC11000839

[6] Ng R. Post-Traumatic transient cortical blindness in a child with occipital bone fracture. J Clin Neurosci. (2016) 34:225–7. 10.1016/j.jocn.2016.08.010 27707613

[7] de Lara J Vázquez-Rodríguez J Salgado-Fernández J Calviño-Santos R Vázquez-González N Castro-Beiras A. Transient cortical blindness following cardiac catheterization:: an alarming but infrequent complication with a good prognosis. Rev Esp Cardiol. (2008) 61(1):88–90. 10.1016/S1885-5857(08)60074-2 18221698

[8] Akhtar N Khatri I Naseer A Ikram J Ahmed W. Transient cortical blindness after coronary angiography: a case report and literature review. J Pak Med Assoc. (2011) 61(3):295–7. 21465952

[9] Yeh S Bazzaz S Foroozan R. Transient cortical blindness with leptomeningeal enhancement after attempted peripherally inserted central venous catheter placement. Arch Ophthalmol. (2005) 123(5):700–2. 10.1001/archopht.123.5.700 15883295

[10] Cascio G Salgia R Shamaa O Montrose J. Transient cortical blindness in the setting of hepatic encephalopathy: an unforeseen outcome following transjugular intrahepatic portosystemic shunt placement. Am J Gastroenterol. (2025) 120(10):S7. 10.14309/01.ajg.0001150912.36098.d241041993

[11] Tugcu B Araz-Ersan B Eren G Selçuk H Yigit U. Transient cortical blindness after spinal surgery as initial presenting sign of hereditary thrombophilia. Indian J Ophthalmol. (2013) 61(3):139–U67. 10.4103/0301-4738.97565 23514658 PMC3665051

[12] Vaiano A Valente C De Benedetti G Caramello G. Transient cortical blindness after intradiscal oxygen-ozone therapy. Indian J Ophthalmol. (2016) 64(12):944–6. 10.4103/0301-4738.198858 28112142 PMC5322716

[13] Tasdemir K Evereklioglu C Kaya M. Transient cortical blindness and successful recovery after coronary bypass surgery. Acta Cardiol. (2011) 66(5):661–4. 10.1080/AC.66.5.2131096 22032065

[14] Ioscovich A Nyman D Briskin A Grisaru-Granovsky S. Transient cortical blindness after caesarean hysterectomy for placenta percreta. Int J Obstet Anesth. (2004) 13(4):291–3. 10.1016/j.ijoa.2004.05.002 15477065

[15] Limaye K Jadhav A. Delayed transient cortical blindness from hypoxic ischemic encephalopathy. Am J Med. (2017) 130(9):E391–2. 10.1016/j.amjmed.2017.03.020 28390792

[16] Yip NZW Barbour-Hastie C Barron P McKee JB Kaliaperumal C. Transient cortical blindness following occipital lobe retraction in a pineal region meningioma resection. BMJ Case Rep. (2025) 18(6):e264865. 10.1136/bcr-2025-264865 40579192 PMC12206958

[17] Schouten D de Graaf S Verrips A. Transient cortical blindness following vincristine therapy. Med Pediatr Oncol. (2003) 41(5):470. 10.1002/mpo.10377 14515391

