# Correction: Transient cortical blindness following coronary angiography: a case report

**DOI:** 10.3389/fcvm.2026.1813052

**Published:** 2026-03-09

**Authors:** Ying Cai, Shengyu Huang, Yuling Jing, Ruijie Ren, Zaiyong Zheng

**Affiliations:** 1 Cardiovascular Center, Chongqing Hospital of Jiangsu Province Hospital, Chongqing, China; 2 Department of Cardiology, The Affiliated Hospital, Southwest Medical University, Luzhou, China; 3 Department of Ultrasound, Chongqing Hospital of Jiangsu Province Hospital, Chongqing, China; 4 Department of Cardiology, Panzhihua Central Hospital, Panzhihua, China

**Keywords:** case report, contrast medium, coronary angiography, cortical blindness, occipital lobe

An incorrect number was provided for the Scientific Research Project of the Science and Technology Bureau of Qijiang District, Chongqing (2023). The correct number is 2023063.

The original version of this article has been updated.

