# Supplementary figures and images for: Transient cortical blindness following coronary angiography: a case report
Source: Front Cardiovasc Med. 2026 Feb 10;13:1774967. doi: 10.3389/fcvm.2026.1774967 (PMC12929470; doi:10.3389/fcvm.2026.1774967)

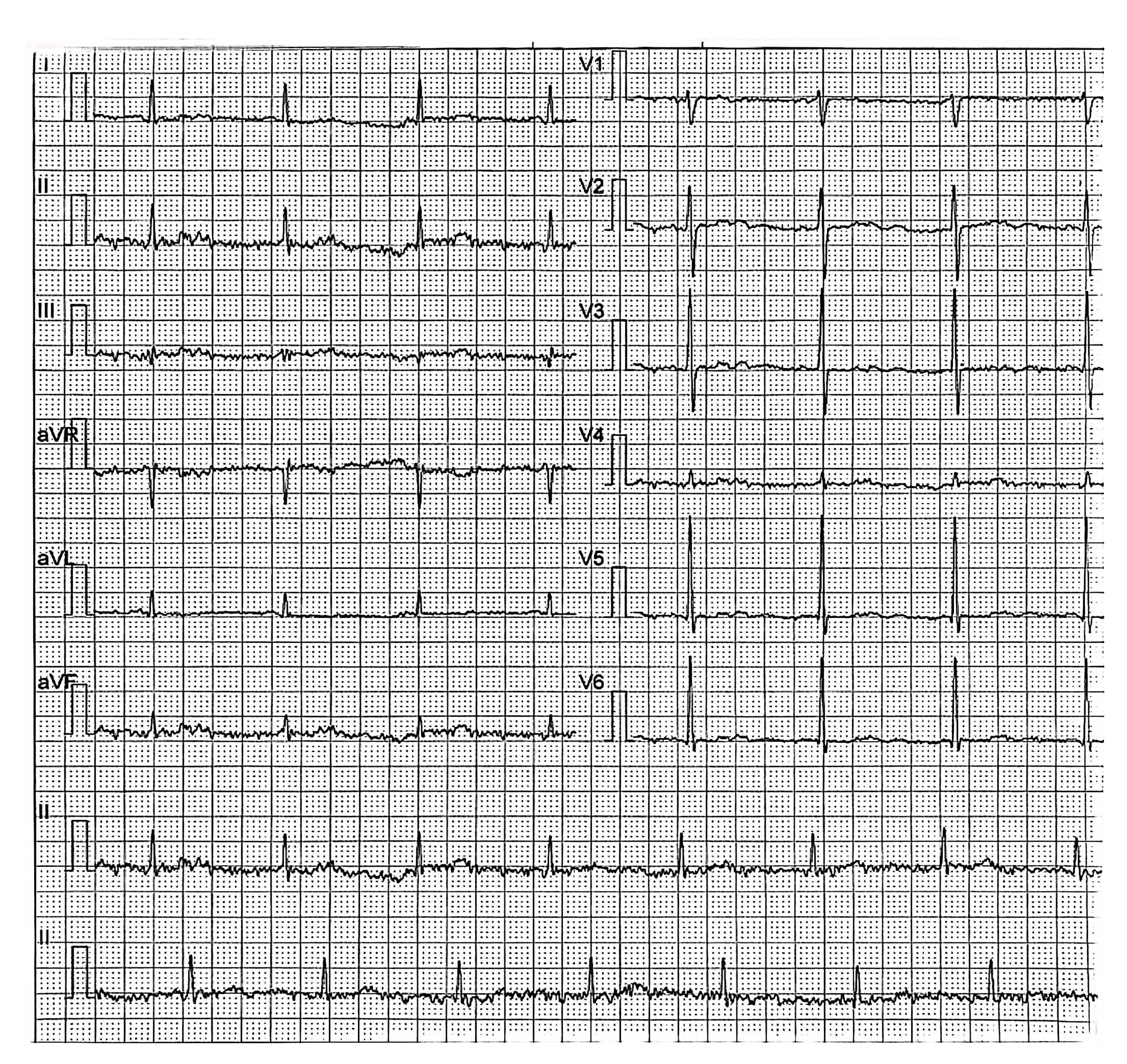

Supplement: Supplementary file 1 [file Image1.tif]
